# Publisher Correction: Unravelling the structure of glycosyl cations via cold-ion infrared spectroscopy

**DOI:** 10.1038/s41467-018-07184-z

**Published:** 2018-11-08

**Authors:** Eike Mucha, Mateusz Marianski, Fei-Fei Xu, Daniel A. Thomas, Gerard Meijer, Gert von Helden, Peter H. Seeberger, Kevin Pagel

**Affiliations:** 1 Department of Molecular Physics, Fritz Haber Institute of the Max Planck Society, Faradayweg 4-6, 14195 Berlin, Germany; 2 Institute of Chemistry and Biochemistry, Freie Universität Berlin, Takustraße 3, 14195 Berlin, Germany; 3 Department of Biomolecular Systems, Max Planck Institute of Colloids and Interfaces, Am Mühlenberg 1, 14476 Potsdam, Germany; 4 Present Address: Hunter College, The City University of New York, 695 Park Ave, New York, NY 10065 USA

Correction to: *Nature Communications* 10.1038/s41467-018-06764-3; published online 09 October 2018

The original version of this Article contained an error in Fig. 1, in which an oxygen atom was missing from the ‘Acetoxonium type’ structure. The correct version of Fig. 1 is:

**Figure 1:**
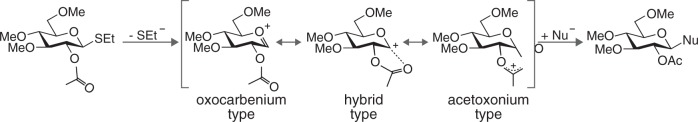
Fig. 1

which replaces the previous incorrect version shown below as Fig. 2:

**Figure 2:**
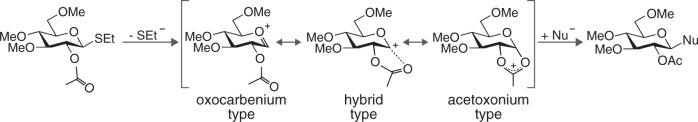
Fig. 2

This has been corrected in both the PDF and HTML versions of the Article.

