# Supplementary material for: Unravelling the structure of glycosyl cations via cold-ion infrared spectroscopy
Source: Nat Commun. 2018 Oct 9;9:4174. doi: 10.1038/s41467-018-06764-3 (PMC6177480; doi:10.1038/s41467-018-06764-3)
Supplement: Supplementary file 1 — Supplementary Information [file 41467_2018_6764_MOESM1_ESM.pdf]

Supplementary Information

Unravelling the Structure of Glycosyl Cations via Cold-Ion Infrared Spectroscopy

Eike Mucha[1,2,†], Mateusz Marianski[1,†,‡], Fei-Fei Xu[3], Daniel A. Thomas[1], Gerard Meijer[1], Gert von Helden[1], Peter H. Seeberger[2,3,*], Kevin Pagel[1,2,*]

[1] Fritz Haber Institute of the Max Planck Society, Department of Molecular Physics, Faradayweg 4-6, 14195 Berlin, Germany

[2] Institute of Chemistry and Biochemistry, Freie Universität Berlin, Takustraße, 14195 Berlin, Germany

[3] Max Planck Institute of Colloids and Interfaces, Department of Biomolecular Systems, Am Mühlenberg 1, 14476 Potsdam, Germany

‡ currently at Hunter College, The City University of New York

† Contributed equally

Correspondence to: kevin.pagel@fu-berlin.de
peter.seeberger@mpikg.mpg.de

Supplementary Note 1

Chemicals were purchased as reagent grade and used without further purification unless stated otherwise. Anhydrous solvents were obtained from Waters Dry Solvent systems. Reactions were monitored by thin-layer chromatography (TLC) analysis, which was visualized by UV light (254 nm) and TLC sugar stain (1% (v/v) 3-methoxyphenol, 30% (v/v) sulfuric acid in ethanol). Flash column chromatography was performed on Kieselgel 60 with 230-400 mesh (Sigma-Aldrich, St. Louis, USA). ^{1}H-NMR, ^{13}C-NMR spectra were recorded on a 400 MHz Varian spectrometer at room temperature. Chemical shifts (in ppm) were calibrated with the solvent residual peak. Coupling constants (J) are reported in Hertz (Hz). Optical rotations (OR) were measured with a Schmidt & Haensch UniPol L 1000 at 589 nm and concentration (c) expressed in g/100 mL. High resolution mass spectrometry (HRMS) was performed on a Waters Xevo Q-Tof mass spectrometer.

3,4,6-Tri-*O*-methyl-α-D-glucopyranose 1,2-(methyl orthoacetate)[1] (13)

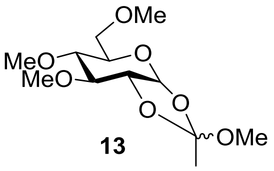

13

MeONa (8.9 mg, 0.16 mmol) was added to a solution of 3,4,6-tri-*O*-acetyl-α-D-glucopyranose 1,2-(methyl orthoacetate) **12** (200 mg, 0.55 mmol) in methanol (4 mL). The reaction mixture was stirred at room temperature for 2 h. After removal of solvent, the residue was dissolved in DMF (4 mL) followed by the addition of sodium hydride (132 mg, 3.3 mmol, 60% wt) at 0 °C. The reaction was stirred for 15 min at room temperature. MeI (0.21 mL, 3.3 mmol) was added and the mixture was then stirred for 2 h. The reaction mixture was quenched with aq. NH_4Cl and extracted with ethyl acetate for three times. The combined organic layer was washed with brine, dried over Na_2SO_4 and concentrated. The residue was purified by flash chromatography with 25% ethyl acetate in hexanes to give product **13** (113 mg, 0.406 mmol, 74%).

$[\alpha]_D^{25}$ +103.94 (c 2.02, $CHCl_3$); ^{1}H NMR (400 MHz, $CDCl_3$) δ 5.69 (d, *J* = 5.2 Hz, 1H), 4.37 (ddd, *J* = 5.3, 3.2, 1.0 Hz, 1H), 3.70 – 3.64 (m, 1H), 3.62 – 3.56 (m, 3H), 3.48 (s, 3H), 3.45 (s, 3H), 3.40 (s, 3H), 3.33 – 3.26 (m, 4H), 1.67 (s, 3H); ^{13}C NMR (101 MHz, $CDCl_3$) δ 121.3, 97.6, 79.7, 77.0, 74.4, 72.2, 69.8, 59.4, 58.4, 57.9, 50.9, 20.7; HRMS (ESI) calcd. for $C_{12}H_{22}O_7Na$ $[M+Na]^+$ 301.1258; found: 301.1244.

Ethyl 2-*O*-acetyl-3,4,6-tri-*O*-methyl-1-thio-β-D-glucopyranoside (2)

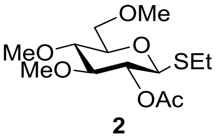

2

Ethanethiol (0.13 mL, 1.8 mmol) and 4 Å molecular sieves were added to a solution of compound **13** (20 mg, 0.072 mmol) in dichloromethane (3 mL). The mixture was stirred for 20 min at room temperature and then cooled down to 0 °C followed by the addition of trimethylsilyl triflate (2.6 μL, 14 μmol). After 2 h, triethylamine was added to quench the reaction. The mixture was diluted with dichloromethane and washed with aq. $NaHCO_3$, brine, dried over Na_2SO_4 and concentrated. The residue was purified by flash chromatography with 20% ethyl acetate in hexanes to give product **2** (18.5 mg, 0.06 mmol, 83%).

$[\alpha]_D^{25}$ -11.0 (c 1.12, $CHCl_3$); ^{1}H NMR (400 MHz, $CDCl_3$) δ 4.88 (dd, *J* = 10.0, 8.8 Hz, 1H), 4.31 (d, *J* = 10.1 Hz, 1H), 3.66 – 3.58 (m, 2H), 3.53 (s, 6H), 3.39 (s, 3H), 3.36 – 3.21 (m, 3H), 2.75 – 2.61 (m, 2H), 2.11 (s, 3H), 1.23 (t, *J* = 7.4 Hz, 3H); ^{13}C NMR (101 MHz, $CDCl_3$) δ 169.8, 86.2, 83.5, 79.4, 79.3, 71.7, 71.5, 60.7, 60.6, 59.5, 24.0, 21.2, 14.9; HRMS (ESI) calcd. for $C_{13}H_{24}O_6SNa$ $[M+Na]^+$ 331.1185; found: 331.1171.

3,4,6-Tri-*O*-methyl-α-D-mannopyranose 1,2-(methyl orthoacetate)[2] (15)

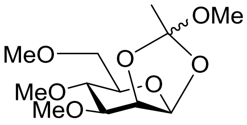

15

MeONa (8.9 mg, 0.16 mmol) was added to a solution of 3,4,6-tri-*O*-acetyl-α-D-mannopyranose 1,2-(methyl orthoacetate) **14** (200 mg, 0.55 mmol) in methanol (4 mL). The reaction mixture was stirred at room temperature for 2 h. After removal of solvent, the residue was dissolved in DMF (4 mL) followed by the addition of sodium hydride (132 mg, 3.3 mmol, 60% wt) at 0 °C. The reaction was stirred for 15 min at room temperature and MeI (0.21 mL, 3.3 mmol) was added. After 2 h, the reaction was quenched with aq. NH_4Cl and extracted with ethyl acetate for three times. The combined organic layer was washed with brine, dried over Na_2SO_4 and concentrated. The residue was purified by flash chromatography with 25% ethyl acetate in hexanes to give product **15** (138 mg, 0.496 mmol, 90%).

$[\alpha]_D^{25}$ -3.18 (c 2.24, $CHCl_3$); ^{1}H NMR (400 MHz, $CDCl_3$) δ 5.40 (d, *J* = 2.6 Hz, 1H), 4.56 (t, *J* = 2.7 Hz, 1H), 3.62 – 3.59 (m, 2H), 3.57 (s, 3H), 3.54 (s, 3H), 3.45 – 3.41 (m, 2H), 3.40 (s, 3H), 3.35 – 3.28 (m, 4H), 1.71 (s, 3H); ^{13}C NMR (101 MHz, $CDCl_3$) δ 124.0, 97.7, 81.4, 76.3, 76.0, 74.1, 71.7, 60.8, 59.5, 58.1, 50.0, 24.4; HRMS (ESI) calcd. for $C_{12}H_{22}O_7Na$ $[M+Na]^+$ 301.1258; found: 301.1246.

Ethyl 2-*O*-acetyl-3,4,6-tri-*O*-methyl-1-thio-α-D-mannopyranose (6)

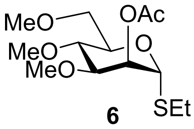

6

Ethanethiol (0.13 mL, 1.8 mmol) and 4 Å molecular sieves were added to a solution of compound **15** (20 mg, 0.072 mmol) in dichloromethane (3 mL). The mixture was stirred for 20 min at room temperature and then cooled down to 0 °C followed by the addition of trimethylsilyl triflate (2.6 µL, 14 µmol). After 2 h, triethylamine was added to quench the reaction. The mixture was diluted with dichloromethane and washed with aq. $NaHCO_3$, brine, dried over Na_2SO_4 and concentrated. The residue was purified by flash chromatography with 20% ethyl acetate in hexanes to give product **6** (11.2 mg, 0.036 mmol, 51%).

$[\alpha]_D^{25}$ +103.2 (c 0.33, $CHCl_3$); ^{1}H NMR (400 MHz, $CDCl_3$) δ 5.34 (t, *J* = 1.9 Hz, 1H), 5.27 (d, *J* = 1.9 Hz, 1H), 3.99 (qd, *J* = 4.3, 1.8 Hz, 1H), 3.66 (dd, *J* = 10.6, 4.3 Hz, 1H), 3.57 (dd, *J* = 10.5, 2.0 Hz, 1H), 3.53 (s, 3H), 3.52 – 3.49 (m, 2H), 3.41 (s, 3H), 3.40 (s, 3H), 2.71 – 2.52 (m, 2H), 2.14 (s, 3H), 1.27 (d, *J* = 8.0 Hz, 3H); ^{13}C NMR (101 MHz, $CDCl_3$) δ 170.6, 80.4, 76.2, 71.5, 71.3, 70.3, 60.9, 59.3, 57.7, 25.6, 21.3, 15.0; HRMS (ESI) calcd. for $C_{13}H_{24}O_6SNa$ $[M+Na]^+$ 331.1185; found: 331.1170.

3,4,6-Tri-*O*-methyl-α-D-galactopyranose 1,2-(methyl orthoacetate) (17)

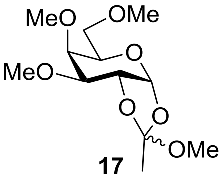

17

MeONa (8.9 mg, 0.16 mmol) was added to a solution of 3,4,6-tri-*O*-acetyl-α-D-galactopyranose 1,2-(methyl orthoacetate) **16** (200 mg, 0.55 mmol) in methanol (4 mL). The reaction mixture was stirred at room temperature for 2 h. After removal of solvent, the residue was dissolved in DMF (4 mL) followed by the addition of sodium hydride (132 mg, 3.3 mmol, 60% wt) at 0 °C. The reaction was stirred for 15 min at room temperature and MeI (0.21 mL, 3.3 mmol) was added.

After 2 h, the reaction was quenched with aq. NH_4Cl and extracted with ethyl acetate for three times. The combined organic layer was washed with brine, dried over Na_2SO_4 and concentrated. The residue was purified by flash chromatography with 30% ethyl acetate in hexanes to afford product **17** (142.8 mg, 0.513 mmol, 93%).

$[\alpha]_D^{25}$ +95.8 (c 1.02, $CHCl_3$); ^1H NMR (400 MHz, $CDCl_3$) δ 5.68 (d, *J* = 4.5 Hz, 1H), 4.30 (dd, *J* = 6.2, 4.5 Hz, 1H), 4.00 (td, *J* = 6.6, 2.6 Hz, 1H), 3.72 (t, *J* = 2.6 Hz, 1H), 3.54 – 3.50 (m, 8H), 3.39 (s, 4H), 3.26 (s, 3H), 1.64 (s, 3H); ^{13}C NMR (101 MHz, $CDCl_3$) δ 122.1, 97.6, 82.7, 79.2, 74.6, 73.0, 70.5, 61.1, 59.4, 57.7, 49.8, 24.5; HRMS (ESI) calcd. for $C_{12}H_{22}O_7Na$ $[M+Na]^+$ 301.1257; found: 301.1241.

Ethyl 2-*O*-acetyl-3,4,6-tri-*O*-methyl-1-thio-β-D-galactopyranoside (7)

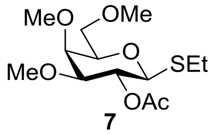

7

Ethanethiol (0.26 mL, 3.6 mmol) and 4 Å molecular sieves were added to a solution of compound **17** (40 mg, 0.14 mmol) in dichloromethane (3 mL). The mixture was stirred for 20 min at room temperature. Then the system was cooled down to 0 ˚C followed by the addition of trimethylsilyl triflate (5.2 μL, 28 μmol). After 2 h, triethylamine was added to quench the reaction. The mixture was diluted with dichloromethane and washed with aq. $NaHCO_3$, brine, dried over Na_2SO_4 and concentrated. The residue was purified by flash chromatography with 20% ethyl acetate in hexanes to obtain product **7** (30.9 mg, 0.10 mmol, 71%) as colorless oil.

$[\alpha]_D^{25}$ -13.2 (c 1.26, $CHCl_3$); ^1H NMR (400 MHz, $CDCl_3$) δ 5.22 (t, *J* = 9.8 Hz, 1H), 4.31 (d, *J* = 9.9 Hz, 1H), 3.74 (d, *J* = 2.9 Hz, 1H), 3.62 – 3.51 (m, 6H), 3.44 (s, 3H), 3.38 (s, 3H), 3.28 (dd, *J* = 9.7, 2.9 Hz, 1H), 2.78 – 2.59 (m, 2H), 2.08 (s, 3H), 1.22 (t, *J* = 7.5 Hz, 3H); ^{13}C NMR (101 MHz, $CDCl_3$) δ 169.9, 83.7, 83.6, 77.5, 77.3, 77.2, 76.8, 74.5, 70.8, 69.5, 61.4, 59.3, 58.0, 23.6, 21.2, 14.8; HRMS (ESI) calcd. for $C_{13}H_{24}O_6SNa$ $[M+Na]^+$ 331.1185; found: 331.1168.

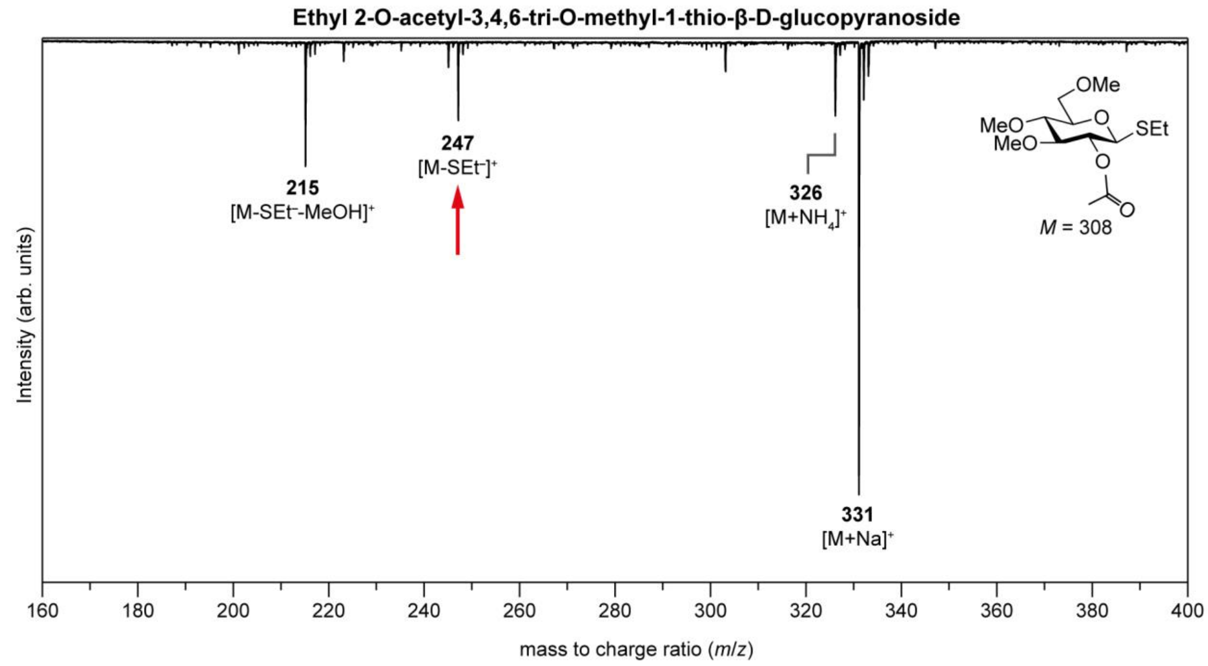

Supplementary Fig. 1. Exemplary mass spectrum of the 2-OAc glucose variant. Glycosyl cations (*m*/*z* = 247) were generated using in-source fragmentation of the respective precursor ions.

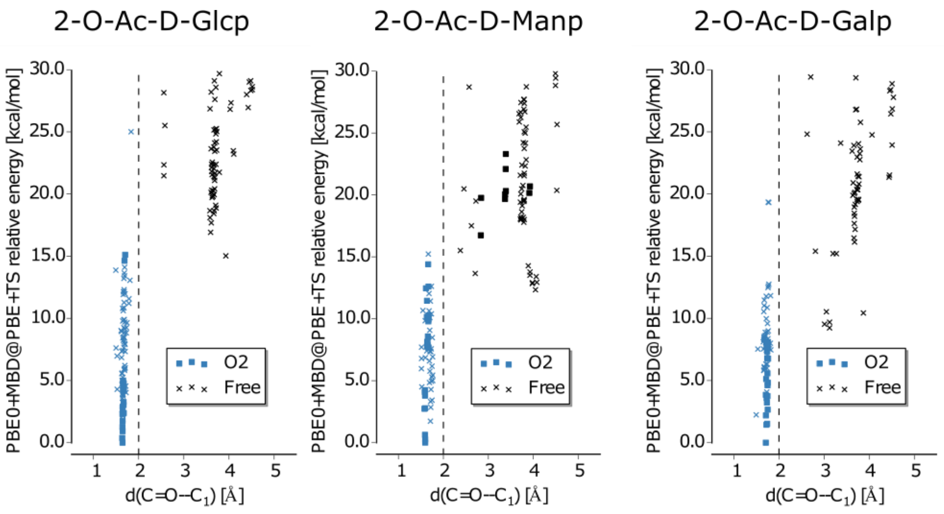

Supplementary Fig. 2. Energy hierarchies. Relative energies calculated as a single-point energy at PBE0+MBD/*tight* at PBE+vdW^{TS}/*light* geometry of all unique carbocation conformations of acetoxonium (blue) and oxocarbenium (grey) structures as a function of C=O – anomeric carbon distance. The square marks highlight ring puckers identical with the lowest energy structure.

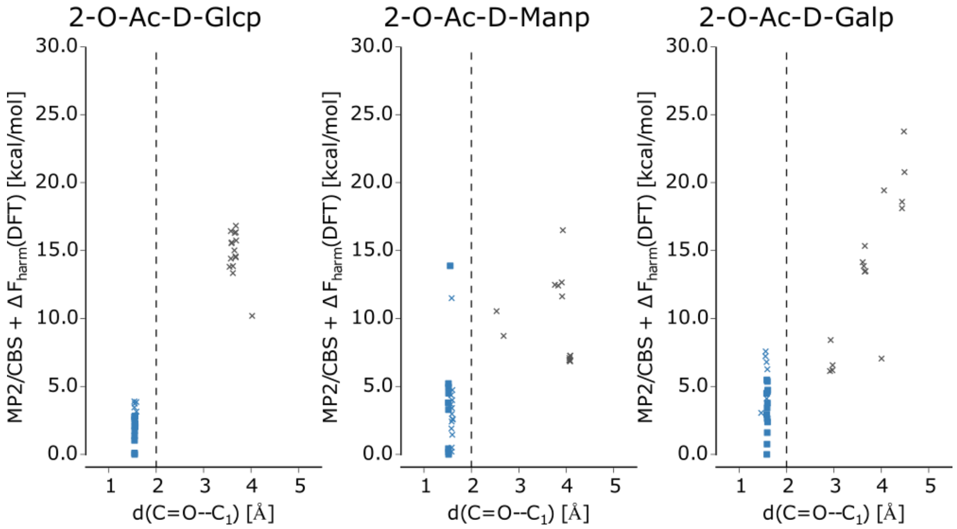

Supplementary Fig. 3. Energy hierarchies. Relative harmonic free-energies of selected carbocation conformations of acetoxonium (blue) and oxocarbenium (grey) structures as a function of C=O – anomeric carbon distance. The geometries has been relaxed at PBE0+D3/6-311+G(d,p) level. Next, the conformational electronic energy was extrapolated to the complete basis set from def2-TVZPP and def2-QVZPP at RI-MP2 level and correction from harmonic free energies at DFT level was added. The square marks highlight ring puckers identical with the lowest energy structure.

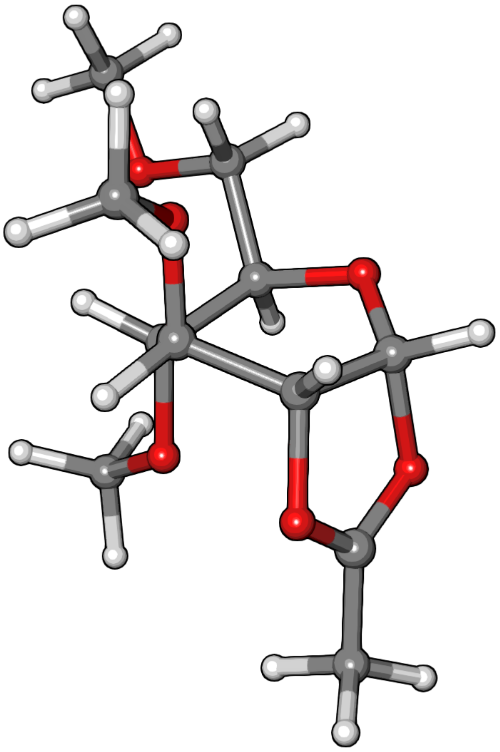

Supplementary Fig. 4. Glucose acetoxonium ion. The lowest-energy structure for the glycosyl cation of the 2-OAc glucose variant adopts a $^{3}S_{1}$ ring pucker.

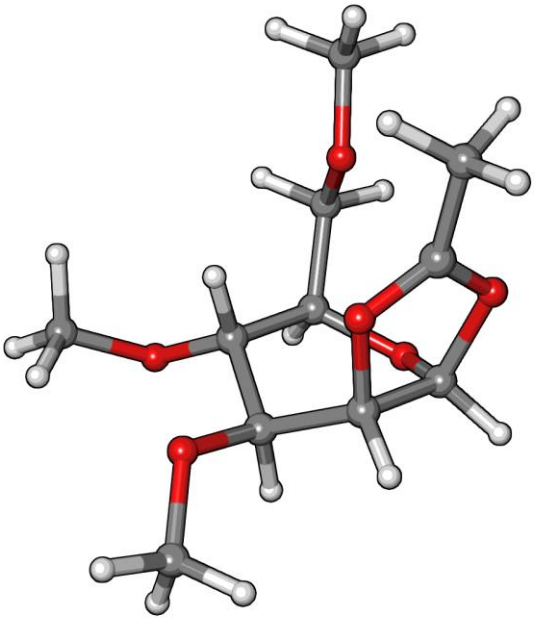

Supplementary Fig. 5. Mannose acetoxonium ion. The lowest-energy structure for the glycosyl cation of the 2-OAc mannose variant adopts a $B_{O,3}$ ring pucker.

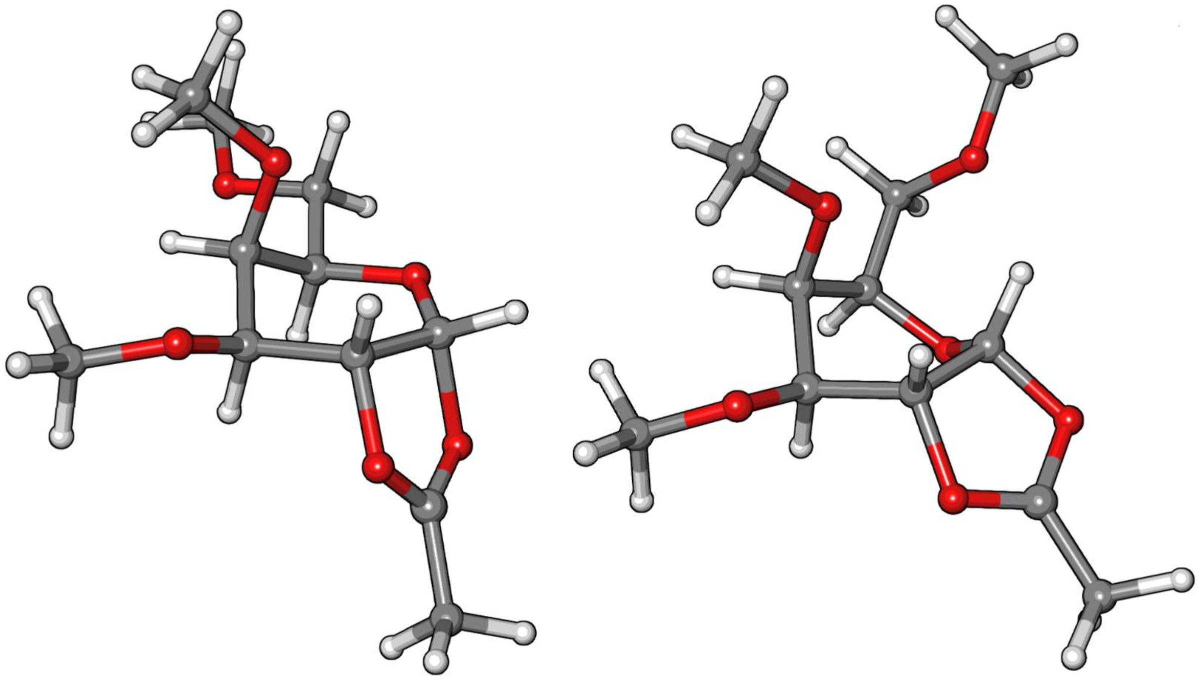

Supplementary Fig. 6. Galactose acetoxonium ions. The lowest-energy structure for the glycosyl cation of the 2-OAc mannose variant adopts a ^{4}E ring pucker (left). The likely co-existing structure exhibits a $^{1}S_{3}$ ring pucker.

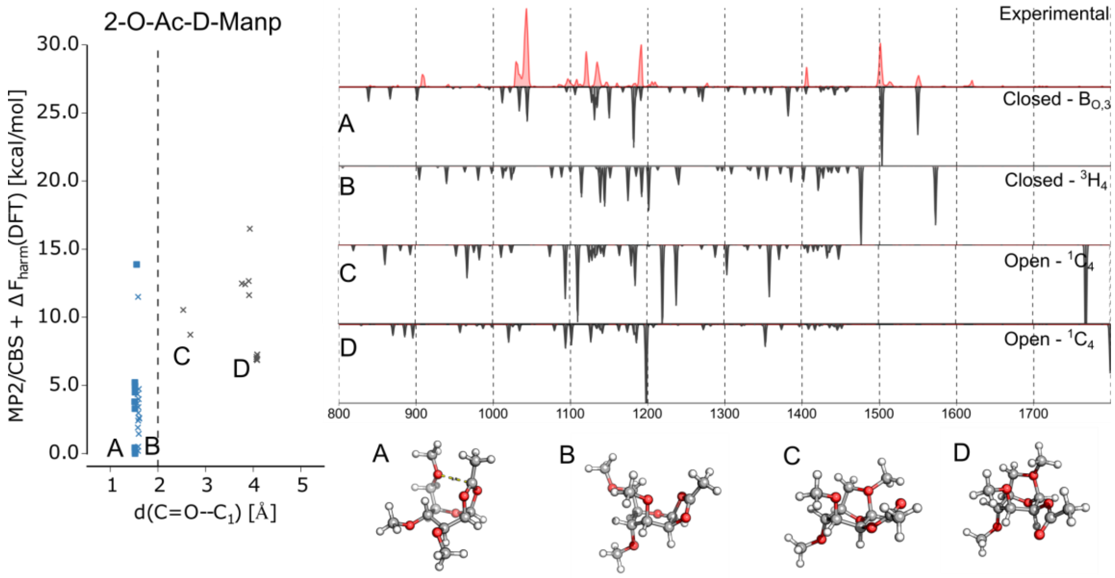

Supplementary Fig. 7. IR Spectra. Exemplary IR spectra of few selected low energy acetoxonium (blue) and oxocarbenium (grey) structures for 2-O-acetyl-D-mannopyranose. The spectra has been calculated at PBE0+D3/6-311+G(d,p) level of theory and are scaled by 0.965 factor.

Supplementary Tab. 1. Bond length. Distances between the carbonyl oxygen of the acetyl group at C2 and the anomeric carbon C1 in Å.

Glycosyl cation	Distance (C–O)–C1 in Å
2-OAc glucose	1.55
2-OAc mannose	1.52
2-OAc galactose	1.58

Supplementary Tab. 2. Parameters. GA parameters used in initial search.

	Parameter	Value
Molecule	Distance_cutoff_1	1.2
	Distance_cutoff_2	2.15
	Rmsd_cutoff_uniq	0.25
GA settings	Popsize	10
	Prob_for_crossing	0.95
	Prob_for_mut_pyranosering	0.5
	Prob_for_mut_torsion_	0.8
	Fitness_sum_limit	1.2
	Selection	Roulette wheel
	Max_mutations_torsion	3

Supplementary Tab. 3. Number of selected structures. Total number of structures at different steps:

Glycosyl cation	Total GA structures	Unique structures	Selected oxocarbenium-type	Selected acetoxonium-type
2-OAc glucose	687	208	15	20
2-OAc mannose	690	170	16	21
2-OAc galactose	684	171	15	20

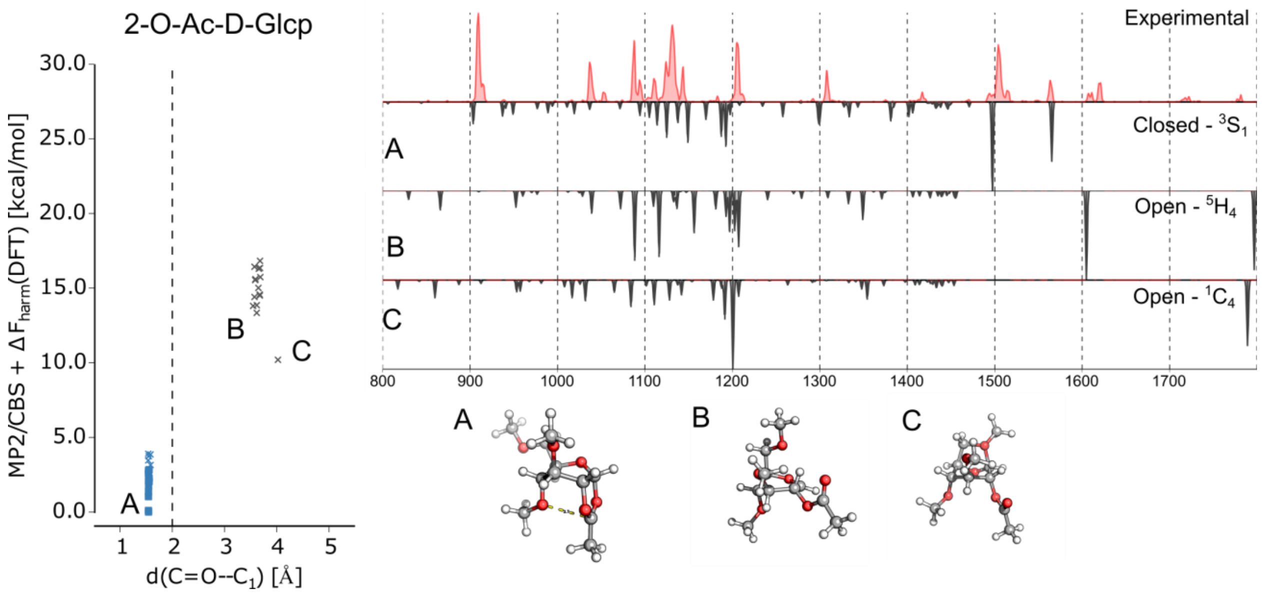

Supplementary Fig. 8. IR Spectra. Exemplary IR spectra of a few selected low energy acetoxonium (blue) and oxocarbenium (grey) structures for 2-O-acetyl-D-glucopyranose. The spectra has been calculated at PBE0+D3/6-311+G(d,p) level of theory and are scaled by 0.965 factor.

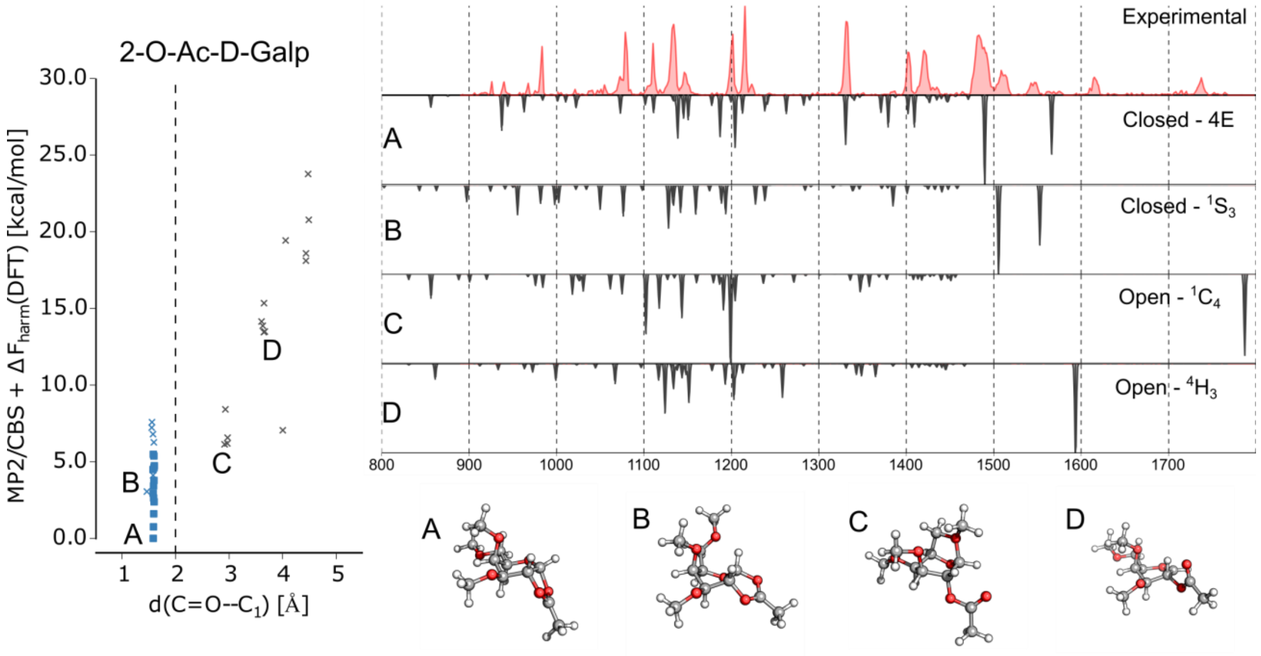

Supplementary Fig. 9. IR Spectra. Exemplary IR spectra of few selected low energy acetoxonium (blue) and oxocarbenium (grey)structures for 2-O-acetyl-D-galactopyranose. The spectra has been calculated at PBE0+D3/6-311+G(d,p) level of theory and are scaled by 0.965 factor.

References

[1] Lin, Y. H., Ghosh, B. & Tony Mong, K.-K. *Chem. Commun.* **48**, 10910-10912 (2012).

[2] Bhattacharjee, S. S. & Gorin, P. A. J. *Carbohydr. Res.* **12**, 57-68 (1970).
